# Primary Metabolite Adjustments Associated With Pinewood Nematode Resistance in *Pinus pinaster*

**DOI:** 10.3389/fpls.2021.777681

**Published:** 2021-11-24

**Authors:** Ana M. Rodrigues, Isabel Carrasquinho, Carla António

**Affiliations:** ^1^Plant Metabolomics Laboratory, Instituto de Tecnologia Química e Biológica António Xavier, Universidade Nova de Lisboa, Oeiras, Portugal; ^2^Instituto Nacional Investigação Agrária e Veterinária I.P., Oeiras, Portugal; ^3^Linking Landscape, Environment, Agriculture and Food, Instituto Superior de Agronomia, Universidade de Lisboa, Lisbon, Portugal

**Keywords:** pinewood nematode (*Bursaphelenchus xylophilus*), maritime pine (*Pinus pinaster*), forest tree metabolomics, pine wilt disease (PWD), primary metabolism, chlorophyll *a* fluorescence (OJIP), plant resistance

## Abstract

The pinewood nematode (PWN) *Bursaphelenchus xylophilus* is the causal agent of the pine wilt disease (PWD) and represents one of the major threats to conifer forests. The detection of the PWN in Portugal, associated with *Pinus pinaster*, increased the concern of its spread to European forests. Despite its susceptibility to PWD, genetic variability found among *P. pinaster* populations has been associated with heritable PWD resistance. Understanding the mechanisms underlying tree resistance constitutes a valuable resource for breeding programs toward more resilient forest plantations. This study investigated changes in anatomy, chlorophyll *a* fluorescence (ChlF), and primary metabolism in susceptible and resistant *P. pinaster* half-sib plants, after PWN inoculation. Susceptible plants showed a general shutdown of central metabolism, osmolyte accumulation, photosynthetic inhibition, and a decrease in the plant water status. The ChlF transient rise (OJIP curve) revealed the appearance of L- and K-bands, indicators of environmental stress. In contrast, resistant plants revealed a regulated defense response and were able to restrict PWN migration and cellular damage. Furthermore, the accumulation of γ-aminobutyric acid (GABA) and succinate suggested a role of these metabolites in PWD resistance and the possible activation of the GABA shunt. Altogether, these results provide new insights to the role of primary metabolism in PWD resistance and in the selection of resistant phenotypes for disease mitigation.

## Introduction

The pinewood nematode (PWN) *Bursaphelenchus xylophilus* is the causal agent of the pine wilt disease (PWD), and represents a major threat to worldwide conifer forests, especially *Pinus* spp., with significant ecological and economical losses. The PWN is vectored by long-horned beetles (*Monochamus* spp.) and spread into healthy trees during maturation feeding or into decaying trees through oviposition (Evans et al., 1996, 2008). In general, PWD symptoms include needle chlorosis and wilting that reflect the blockage of the water-conducting system caused by cavitation and embolism of the tracheids in the xylem, leading to rapid tree desiccation and death (Myers, 1988; Fukuda, 1997; Futai, 2013).

The PWN is native to North America and was introduced in Japan in 1905 where the PWD was first reported in Japanese red pine (*Pinus densiflora*) (Futai, 2008). It then spread to other East Asian countries devastating other native pine species, such as *Pinus thunbergii* and *Pinus massoniana* (Nose and Shiraishi, 2008). Human activities (e.g., international wood trade) promoted the geographic expansion of the PWN, and in 1999 it was detected in Portugal associated with maritime pine (*Pinus pinaster*) (Mota et al., 1999). Since then, the PWN has reached Spanish border territories, thereby increasing the concern of its spread to European forests (Abelleira et al., 2011).

The PWN is classified by the European and Mediterranean Plant Protection Organization as a A2 quarantine pest. As a consequence, the open trade of wood products derived from geographic areas where its occurrence is documented is highly regulated (EPPO, 2018). To control the presence of the PWN and prevent its spread to other European territories, current phytosanitary safety measures mainly include the identification and elimination of symptomatic trees, the creation of a buffer zone across the Portugal-Spain border, and the control of the insect vector population (Rodrigues, 2008; ICNF, 2019). However, the expansion of the PWD to European forests is projected to be aggravated in the current global climate change scenario, due to the intensity and frequency of adverse environmental-stress factors, such as increased temperatures and drought events (Hirata et al., 2017; de la Fuente and Beck, 2018; de la Fuente et al., 2018; Ikegami and Jenkins, 2018; Gruffudd et al., 2019).

Breeding for resistance is one relevant forest management strategy to control pest and disease outbreaks and promote the health of forest ecosystems that are under increasing environmental stress. The implementation of mass selection breeding programs is crucial to generate populations with quantitative resistance and accelerate the widespread dissemination of resistant genotypes (Ennos, 2015). Intraspecific variation in PWD resistance within susceptible species prompted the launch of breeding programs in Asian countries affected by the disease; namely, Japan in 1978 for the selection of resistant *P. densiflora* and *P. thunbergii* trees, and China in 2001 for resistant *P. massoniana* (Nose and Shiraishi, 2008). In 2009, a similar resistance breeding program for *P. pinaster* was initiated in Portugal (Ribeiro, 2012). From a reference population of 457 *P. pinaster* plus trees, 96 half-sib families were tested for genetic survival variability to PWN infection and the 15 top-ranked families were selected for clonal seed orchard establishment (Carrasquinho et al., 2018). However, resistance to PWN infection is a quantitative trait, and within a half-sib family, only a small fraction of the individuals may prove resistant while the majority are susceptible. For the selected 96 *P. pinaster* half-sib families, predicted survival means at 157 days after inoculation ranged from 7 to 23% (Carrasquinho et al., 2018). *Pinus pinaster* is a native species from the western Mediterranean basin, but its distribution is greatly fragmented. Studies on molecular markers and quantitative traits have shown high genetic variability in morphological and adaptative traits among populations from different provenances (González-Martínez et al., 2002). This genetic variability has been further correlated with significant differences in susceptibility to PWN infection among different *P. pinaster* populations from the Iberian Peninsula and France (Zas et al., 2015; Menéndez-Gutiérrez et al., 2017).

Despite the numerous studies on PWN and PWD, the current knowledge on the molecular mechanisms underlying tree resistance to PWD is still very limited. In addition, only few studies have focused on the comparison of transcriptional responses between resistant and susceptible plants, within susceptible species, namely in *P. thunbergii* (Hirao et al., 2012), *P. massoniana* (Liu et al., 2017) and more recently *P. pinaster* (Modesto et al., 2021). These studies showed that resistance and susceptibility to PWD do not rely only on qualitative differences in gene expression, but also on variations in the timing and magnitude of their expression. In general, the transcriptome profiling of *Pinus* spp. after PWN infection has revealed differentially expressed genes mostly related to secondary metabolism (e.g., phytohormone, phenylpropanoid, and terpenoid biosynthesis), ROS detoxification, and also to primary metabolism, namely carbohydrate metabolism (Gaspar et al., 2017, 2020; Liu et al., 2017; Lee et al., 2018, 2019; Modesto et al., 2021). Alterations in secondary metabolism in response PWN infection include an increase in defense-related phytohormones involved in jasmonic acid (JA) and salicylic acid (SA) pathways in PWN-susceptible *P. pinaster*, but not in resistant phenotypes (Modesto et al., 2021; Rodrigues et al., 2021). On the other hand, transcriptomic profiling of *P. pinaster* plants revealed an enrichment in amino sugar and nucleotide sugar metabolic pathways only in resistant plants (Modesto et al., 2021). The regulation of primary metabolism in biotic stress responses has gained increasing interest, mainly due to its key role in fulfilling the energetic demands for plant defense mechanisms and as a source of signaling molecules (Berger et al., 2007; Bolton, 2009; Steinbrenner et al., 2011; Rojas et al., 2014). Thus, following the recent advances in transcriptomic analysis, a metabolomics approach would provide comprehensive information on the metabolite profiling of pine tree tissues in response to PWN infection, as it is considered the molecular phenotype of a living organism, reflecting the interaction between the genetic traits and environmental factors (Fiehn, 2002; Fernie et al., 2004).

In this study, a gas chromatography coupled to time-of-flight mass spectrometry (GC-TOF-MS) platform was used to characterize the primary metabolome of a *P. pinaster* half-sib family, after PWN inoculation. This family has been previously genetically evaluated for PWD resistance in a mass selection program using Empirical Best Linear Unbiased Prediction (EBLUP) and estimated Breeding Values (Carrasquinho et al., 2018). Primary metabolite profiling of susceptible and resistant plants was further integrated with morphological, physiological and anatomical parameters for a comprehensive characterization of *P. pinaster* differential response to PWN infection. This integrative approach can allow the identification of potential primary metabolism-related resistance traits that can be further used in future studies for PWD management.

## Materials and Methods

### Plant Material

*Pinus pinaster* Aiton seeds were obtained from a plus tree (half-sib family 440) belonging to a breeding population for PWD resistance at “Herdade da Comporta” (38°21′28.52″N; 8°45′49.89″W) in southern Portugal (Ribeiro, 2012). This half-sib family was classified as one of the 15 top-ranked half-sib families (out of 96) regarding the resistance to PWD (Carrasquinho et al., 2018). After cold-wet stratification (three weeks in the dark at 4°C), stratified seeds were germinated in forestry trays (54-universal, Sinorplásticos, Espinho, Portugal) in greenhouse conditions. Plants were grown for 22 months under natural daylight in a greenhouse equipped with a cooling system and an automatic sprinkler irrigation system set for 5 min/48 h during the winter and 3 min/24 h during the summer.

### Pinewood Nematode Inoculum

*Bursaphelenchus xylophilus* (Steiner and Buhrer) Nickle isolate Bx013.003 used in this study was obtained from an infected field tree exhibiting PWD symptoms in central Portugal (39°43′33.8″N, 9°01′55.7″W) and was included in the nematode collection of the Nematology Laboratory at INIAV (Oeiras, Portugal). The sequence of the internal transcribed spacer (ITS) region is available at GenBank database (NCBI) under the accession number MF611984.1. Nematodes were extracted using the “tray” method (Whitehead and Hemming, 1965) and maintained in cultures of a non-sporulating *Botrytis cinerea* strain grown on steam-sterilized hydrated barley (*Hordeum vulgare*) grains, at 25 ± 1^°^C. Prior to inoculation, nematodes were allowed to grow on sterilized wood. Nematodes were isolated from the culture media using the tray method (Whitehead and Hemming, 1965) and suspended in water in a concentration of 1000 PWN/mL.

### Experimental Design

**FIGURE 1:**
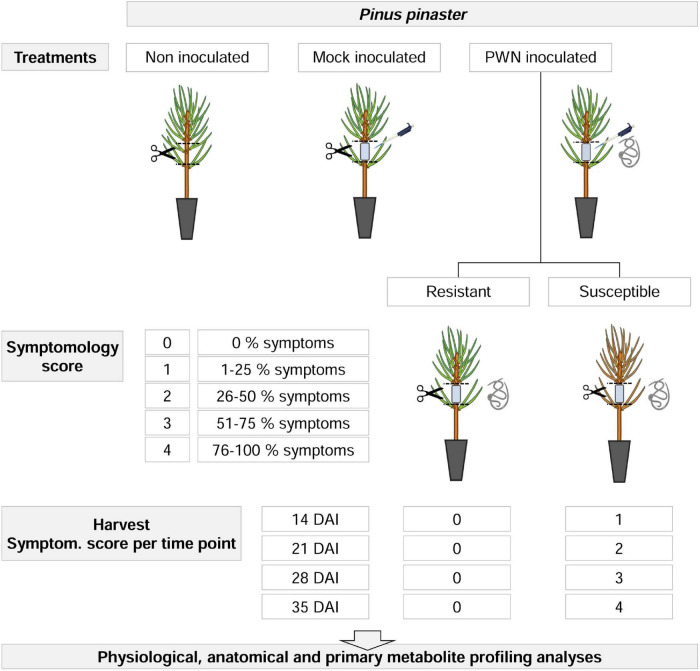
Experimental design for physiological, anatomical and primary metabolite profiling analyses in 22-month-old *Pinus pinaster* half-sib plants, up to 35 days after inoculation (DAI) with the pinewood nematode (PWN) *Bursaphelenchus xylophilus*. First, three manipulative treatments were applied (i.e., non-inoculated, mock-inoculated and PWN-inoculated). For PWN-inoculated plants, a symptomology score was established based on external symptoms development, ranging from 0 (no external symptoms, i.e., resistant plants) to 4 (76–100% external symptoms). At each sampling time point (14, 21, 28, and 35 DAI), PWN-inoculated plants were characterized as resistant or susceptible, according to the symptomology score and harvested as indicated.

The experimental design layout consisted of a completely randomized design, with two factors: inoculation and time after inoculation, with three and four levels, respectively (Figure 1). For inoculation levels were: (i) non-inoculated, (ii) mock-inoculated, and (iii) PWN-inoculated plants; and for time after inoculation levels were: (i) 14, (ii) 21, (iii) 28, and (iv) 35 days after inoculation (DAI), respectively. For a total of 633 plants, the height and basal stem diameter were measured before inoculation, using a marked scale and a digital caliper (Mitutoyo CD-15DCX, Mitutoyo Corp., Kawasaki, Japan).

### Inoculation and Sampling Procedure

For the inoculation process (Supplementary Figure 1), needles were manually removed from an area of ca. 5 cm in the upper part of the stem of each plant. In this area, superficial longitudinal incisions were performed with a sterile razor blade, and a sterilized piece of cotton was placed and fixed with Parafilm^®^. A suspension with an estimated number of 500 PWNs (at various stages of development) was applied with a micropipette, and the cotton was gently covered with Parafilm^®^ to prevent the inoculum from drying. A mock inoculation was performed by replacing the PWN suspension with sterile water.

**FIGURE 2:**
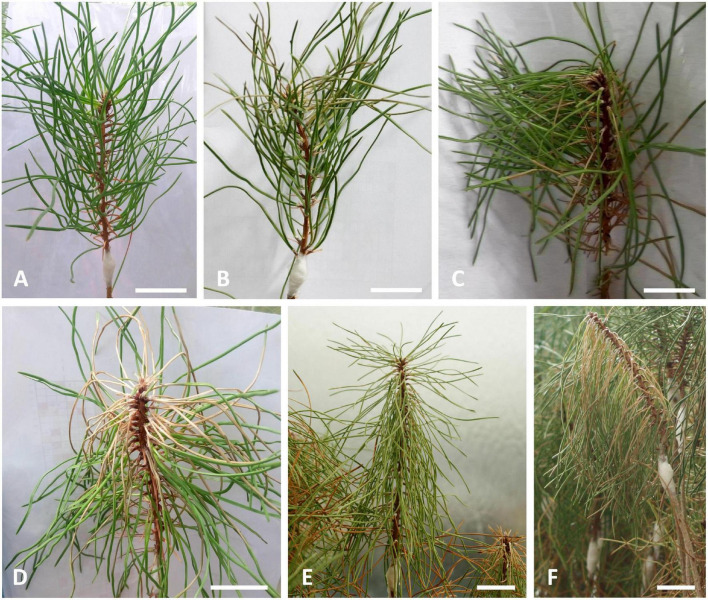
Visual evaluation of external symptoms in 22-month-old *Pinus pinaster* half-sib plants after inoculation with the pinewood nematode (PWN) *Bursaphelenchus xylophilus*, up to 35 days after inoculation (DAI) and according to a symptomology score defined by the percentage of discolored and/or wilting needles, namely **(A)** 0 % (35 DAI), **(B)** 1–25 % (14 DAI), **(C)** 26–50 % (21 DAI), **(D)** 51–75 % (28 DAI), **(E,F)** 76–100 % (35 DAI). Scale bars = 5 cm.

For PWN-inoculated plants, a symptomology score ranging from 0 to 4 was established based on the percentage of discolored and wilted needles; namely, 0 (i.e., no external symptoms), 1 (1–25 %), 2 (26–50 %), 3 (51–75 %), and 4 (76–100 %). At each sampling time point (14, 21, 28, and 35 DAI), PWN-inoculated plants were classified as resistant or susceptible according to the symptomology score, and harvested according to PWD symptom progression as indicated in Figures 1, 2. To assure that metabolomics analysis reflected the PWD symptom progression only plants with score 1 were harvested at 14 DAI; score 2 harvested at 21 DAI; score 3 harvested at 28 DAI; and finally score 4 at 35 DAI. In this study, resistance to PWN infection was considered when inoculated plants showed no external symptoms (i.e., no discolored and/or wilted needles), and were able to limit nematode multiplication and infection (Kover and Schaal, 2002; Pagán and García-Arenal, 2018).

The inoculation area of 15 biological replicates, as well as the corresponding stem portion in non-inoculated plants, were collected, immediately placed in liquid nitrogen, and stored at –80 °C until sample processing for GC-TOF-MS analysis. Needles from each biological replicate were also collected for plant water status characterization. Stem segment samples, from three biological replicates, were immediately placed in buffered glutaraldehyde for microscopy analysis.

During the course of the experiment, the average day/night air temperature and relative humidity was 27/21°C and 63/76 %, respectively.

### Plant Physiological Characterization

#### Plant Water Status

Plant water status was estimated *via* relative water content (RWC) as previously described in Amaral et al. (2019). From each 15 biological replicates, fresh weight (FW) of six needles was measured immediately after collection. Needles were transferred to tubes with distilled water and incubated overnight at 4°C. The excess water was then carefully removed and turgid weight (TW) was registered. Needles were oven-dried at 60 °C for one week and reweighted for dry weight (DW) determination. RWC was calculated as:


$$\text{RWC}\left(\%\right)=\frac{\text{FW}-\text{DW}}{\text{TW}-\text{DW}}\times 100$$

#### Chlorophyll *a* Fluorescence

Chlorophyll *a* fluorescence (ChlF) kinetic measurements were made *in vivo* at 10 am using a Handy Plant Efficiency Analyzer (PEA) – Chlorophyll Fluorimeter (Hansatech Instruments, Kings Lynn, United Kingdom). The needles to be measured (green, non-detached) were arranged in parallel completely covering the 4 mm^2^ test hole, and were dark adapted with light-withholding leaf clips (Hansatech Instruments, Kings Lynn, United Kingdom) for 15 min. Samples were then exposed to a saturating light pulse (3500 μmol m^–2^ s^–1^) for 1 s in order to ensure closure of all PSII reaction centers (RC) and obtain the ChlF transient rise (OJIP curve) (Strasser et al., 2004, 2010; Stirbet and Govindjee, 2011; Goltsev et al., 2016). ChlF intensity at 20 μs was considered as the minimum fluorescence F_0_ (Strasser et al., 2004). Each OJIP curve represents the mean values obtained from 15 biological replicates.

For a better visualization of the effect of PWN inoculation on the transient dynamics, the OJIP curves were plotted as (i) fluorescence intensity; (ii) relative variable fluorescence (V_t_) double normalized to F_0_ and maximum fluorescence F_m_; and (iii) differences in relative fluorescence rise kinetics (ΔV_t_) by subtracting the V_t_ values recorded for non-inoculated control plants from those recorded for mock-inoculated and PWN-inoculated plants.

ChlF transients were analyzed using data extracted from the OJIP-test parameters; namely, (i) normalized derived data; (ii) energy fluxes per active RC and per leaf cross section at fully closed RC (CS_m_) or fully open RC (CS_0_); (iii) quantum yields; (iv) density of RC, and (v) rates and performance indexes (PI). Definitions and derivations of all OJIP-test transients (Strasser et al., 2004, 2010; Stirbet and Govindjee, 2011; Goltsev et al., 2016) are summarized in Supplementary Table 1.

### Gas Chromatography Coupled to Time-of-Flight Mass Spectrometry Primary Metabolite Profiling

Primary metabolites were extracted from 100 mg fresh weight (FW) of finely homogenized stem material per biological replicate (previously macerated under liquid nitrogen), in 1400 μL ice-cold methanol with 60 μL of ribitol (0.2 mg mL^–1^ ribitol in water) as internal standard, as previously described in Lisec et al. (2006). The mixtures were vortex-mixed and incubated in a shaker (ThermoMixer, Eppendorf, Hamburg, Germany) for 15 min at 70°C and 950 rpm. After centrifugation at room temperature and 12000 *g* for 10 min, the supernatant was collected, mixed with 750 μL chloroform and 1500 μL water, and vortex-mixed. The mixtures were centrifuged again at 2200 *g* for 15 min, and 150 μL of the polar (upper) aqueous/methanol phase were evaporated to dryness using a centrifugal concentrator for a minimum of 3 h at 30°C (Vacufuge Plus, Eppendorf, Hamburg, Germany), and stored at –80°C.

Primary metabolites were derivatized using methoxyamination and trimethylsilylating reagents and analyzed as in Lisec et al. (2006). Briefly, dried samples were dissolved in 40 μL of methoxyamine hydrochloride (20 mg mL^–1^ in pyridine), and incubated in a shaker for 2 h at 37°C. Next, 70 μL of *N*-methyl-*N*-(trimethylsilyl)trifluoroacetamide (MSTFA) and 20 μL of a mixture of fatty acid methyl esters (FAMEs) were added and shaken for 30 min at 37°C.

Primary metabolite profiling analysis of the derivatized samples (1 μL aliquots) was performed on an Agilent 6890N gas chromatograph (Agilent Technologies, Böblingen, Germany), and a LECO Pegasus III TOF-MS running in electron ionization (EI) mode (LECO Instrumente, Mönchengladbach, Germany). The chromatographic separation was performed on a VF-5MS column (Varian Inc., 30 m length, 0.25 mm inner diameter, and 0.25 μm film thickness). The temperature program was set as follows: isothermal for 2 min at 85°C, followed by a 15°C min^–1^ ramp to 360°C, and hold at this temperature for 6 min. The injector and transfer line temperatures were set to 230°C and 250°C, respectively, and the injection was performed both in the splitless and split (1:5) mode with helium carrier gas flow set to 2 mL min^–1^. After a solvent delay of 180 s, mass spectra were scanned from *m/z* 70–600 with acquisition rate of 20 spectra s^–1^ and a detector voltage between 1700 and 1850 V.

Biological variations were controlled by analyzing FAMEs as retention time index internal standard markers, and a quality control standard solution of 41 pure reference compounds (i.e., the most detected and abundant metabolites) throughout the analysis. GC-TOF-MS data were evaluated using AMDIS (Automated Mass Spectral Deconvolution and Identification System) software (v. 2.71). Primary metabolites were annotated using the TagFinder 4.0 software (Luedemann et al., 2008) and a reference library of ambient mass spectra and retention indices from the Golm Metabolome Database^1^ (Kopka et al., 2005; Schauer et al., 2005). Metadata information of this experiment following minimum reporting standard guidelines of the Metabolomics Standard Initiative (Lisec et al., 2006; Fiehn et al., 2007; Sumner et al., 2007; Fernie et al., 2011; Alseekh et al., 2021) can be found in Supplementary Table 2.

### Histological Characterization

*Pinus pinaster* stem samples were fixed with glutaraldehyde 2.5 % (v/v) in 0.1 M sodium phosphate buffer, pH 7.2. Samples were kept in the fixative under vacuum at room temperature for 30 min, followed by three days at 4°C. The material was then washed in the fixative buffer and dehydrated in a graded series of ethanol before being infiltrated and embedded in Technovit^®^ 7100 resin (Heraeus Kulzer, Wehrheim, Germany), according to the manufacturer‘s instructions. Transversal cross sections (2 μm thick) were cut using a Leica RM 2155 microtome (Leica Microsystems, Nussloch, Germany) and heat-fixed to slides. To highlight the contrast between the plant tissues and PWNs, sections were stained with a periodic acid-Schiff (PAS) staining system (Sigma Aldrich), following manufacturer standard procedure for polysaccharides detection and counter-stained with Coomassie blue stain for proteins (Fisher, 1968). Stained sections were examined under a Leica DM6 B Upright microscope, coupled to a Leica DFC7000 T camera and using the LAS X system of image capture software (Leica Microsystems GmbH, Wetzlar, Germany).

### Statistical Analysis

Statistical analyses were performed in R and R Studio software (RStudio Team, 2018; R Core Team, 2019). R Packages used to perform statistical analysis include “agricolae” (Mendiburu, 2020), “car” (Fox and Weisberg, 2019), “gplots” (Warnes et al., 2020), “ggplot2” (Wickham, 2016), “mixOmics” (Rohart et al., 2017), “olsrr” (Hebbali, 2020), “scales” (Wickham and Seidel, 2020), and “stats” (R Core Team, 2019). Plant height, diameter, physiological and metabolite data were used as response variables. Each time point was analyzed independently as a single factor with four inoculation levels (i.e., non-inoculated, mock inoculated, susceptible PWN-inoculated, and resistant PWN-inoculated plants). The wound effect was assessed by comparing mock-inoculated with non-inoculated controls. PWN infection was evaluated comparing PWN-inoculated with mock-inoculated plants.

To analyze plant survival, a logistic regression, i.e., a generalized linear model with logit link function and binomial error distribution, was fitted. The model considered the effects of plant height and plant diameter as predictors in the survival outcome. Binned residual plot was used to confirm the fit of the regression model.

Physiological data was analyzed using the Kruskal-Wallis non-parametric test, with a False Discovery Rate (FDR) correction on the *p*-values.

For the metabolomics data, the relative abundance of primary metabolite levels was normalized to the internal standard (ribitol) and the fresh weight of the samples. Metabolomics data were log-transformed before statistical analysis to fit the normality and homoscedasticity assumptions of the ANOVA using Shapiro-Wilk and Levene’s test, respectively. For each time point of data collection, one-way ANOVA at a 95 % confidence level was used to assess differences between treatments, with an FDR correction on the p-values for multiple comparisons. In addition, multiple comparison analysis was performed using Tukey’s HSD test.

Supervised Partial Least Squares – Discriminant Analysis (PLS-DA) was performed using the leave-one-out cross-validation embedded in the “mixOmics” package. Sparse PLS (sPLS) was used to integrate physiological and metabolomics data. Specifically, metabolite data were used as predictors for physiological data. The regression models were tuned based on total Q2 for component selection and Mean Square Error for variable selection for each component (Lê Cao et al., 2008).

## Results

### Pine Wilt Disease Progression

After inoculation with the PWN, PWD external symptoms were assessed weekly (Figure 2). At 14 DAI, needles of susceptible plants started to fade to a grayish color (needle discoloration). Symptoms progressed rapidly during the following days as needles wilted and turned to a yellowish and brown colors. At 28 DAI, more than half of the plants scored level 3 or 4; that is, plants exhibited at least 50 % of needle discoloration and/or wilting. At 35 and 42 DAI, 77 % and 92 % of the plants, respectively, showed more than 75 % of needles being desiccated and brown (score level 4). Resistant plants showed no external symptoms of disease progression. Results from the logistic regression model, showed that the effect of plant height and plant diameter on plant survival were not statistically significant (*p* > 0.05), and thus, were not considered as covariates in the subsequent metabolomics and physiological analysis.

### Histological Characterization of Pinewood Nematode-Inoculated *Pinus pinaster* Plants

**FIGURE 3:**
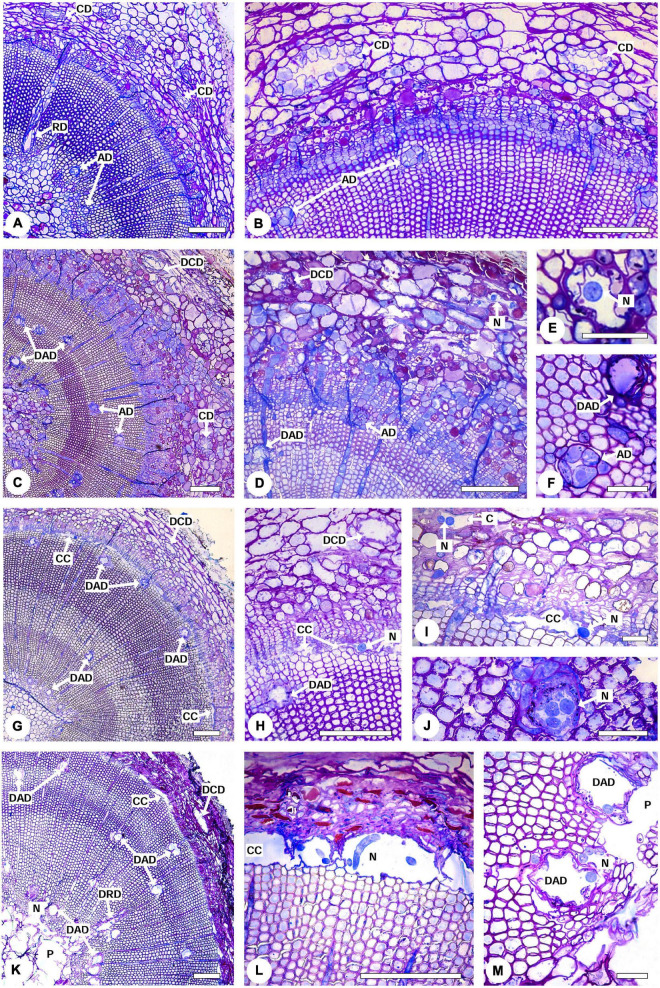
Light micrographs of stem cross-sections from 22-month-old *Pinus pinaster* plants non-inoculated and 39 days after inoculation with the pinewood nematode (PWN) *Bursaphelenchus xylophilus*. **(A,B)** Non-inoculated control plant showing the characteristic anatomy of a pine stem with no signs of cell degradation, namely cortical resin ducts (CD), radial (RD) and axial resin ducts (AD) surrounded by intact epithelial cells. **(C–F)** PWN-inoculated resistant plant showing destruction of the epithelial cells of some cortical and axial resin ducts (DCD and DAD, respectively). Nematodes (N) were observed within cavities in the cortex **(D)** and the lumen of the axial resin ducts **(E)**. **(F)** Detail of an intact (AD) and destroyed (DAD) axial resin duct. **(G–J)** PWN inoculated susceptible plant with 50 % external symptoms showing cavities formed by the degradation of cells in the cambial zone (CC) and destruction of the epithelial cells of the cortical (DCD) and axial (DAD) resin ducts. Nematodes (N) can be observed within cavities in the cambial region **(H)**, cortex **(I)**, and within the axial resin ducts **(J)**. **(K–M)** PWN-inoculated susceptible plant with 100 % external symptoms. The pith tissue was completely destroyed (P), cavities in the cambium enlarged (CC) and nematodes (N) were observed in the pith area **(K)**, cavities in the cambium **(L)** and axial resin ducts **(M)**. Scale bars = 200 μm **(A–D,G,H,K–M)**, 50 μm **(E,F,I,J)**.

Pine wilt disease (PWD) progression and the presence of the PWN in inoculated *P. pinaster* plants was characterized by light microscopy in comparison to controls (Figure 3). The typical anatomy of a pine stem was observed in control plants; namely, several axial resin ducts distributed in the cortex and in the xylem as well as radial resin ducts along the xylem rays extending from the secondary xylem to the cortex with no signs of cell degradation (Figures 3A,B). Resistant plants (i.e., no external symptoms at 35 DAI) showed destroyed epithelial cells in some cortical and axial resin ducts and the formation of cavities in the cortex (Figures 3C–F). Nematodes were observed within these cavities, and in the lumen of the destroyed resin ducts. However, cell degradation in resistant plants was limited to a small area of the stem, most likely the inoculation area. The occlusion of the cortical resin canals by a gel-like resin was also observed in resistant plants (Figure 3F). In susceptible plants with ca. 50 % external symptoms, cortical and axial resin ducts were completely destroyed, and cavities were formed due to the degradation of cells in the cambial zone as well as in the cortical resin ducts and surrounding parenchyma cells (Figure 3G). Nematodes were found within these cavities in the cambial region, cavities in the cortex, and in the degraded axial resin ducts (Figures 3H–J). As the disease progressed in susceptible plants, severe degradation of all stem tissues was visible. Cells in the cortex were completely degraded, the cavities in the cambium and phloem enlarged and fused, and as a result of the migration of the nematodes to the inner tissues, the pith tissue was severely destroyed. Nematodes were found in the pith area, cavities in the cambium, and in the lumen of the axial resin ducts (Figures 3K–M).

### Plant Water Status

**FIGURE 4:**
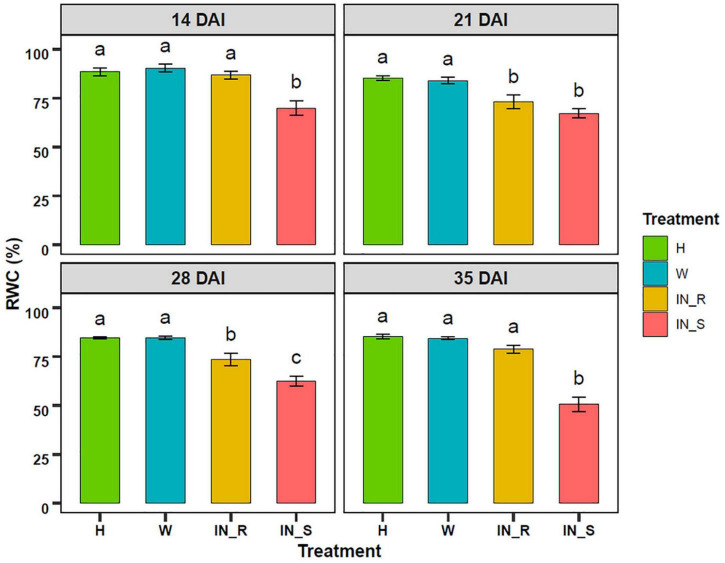
Variation of the relative water content (RWC, %) in needles of 22-month-old *Pinus pinaster* plants 14, 21, 28, and 35 days after inoculation (DAI) with the pinewood nematode *Bursaphelenchus xylophilus*. Bars represent the mean values ± SE from 15 independent measurements. One-way ANOVA (*p* < 0.05) followed by Tukey’s HSD test was performed for means comparison and different letters express significant differences between treatments: non-inoculated (healthy, H), mock-inoculated (wounded, W), resistant PWN-inoculated (IN_R), susceptible PWN-inoculated (IN_S).

Pinewood nematode (PWN) inoculation caused a decrease in the RWC of susceptible plants, in relation to non-inoculated and mock-inoculated control groups (Figure 4). This decrease was more pronounced at 35 DAI, with RWC values reduced to 50 %. Resistant plants showed a significant decrease in the RWC at 21 and 28 DAI. Nevertheless, at 35 DAI, the RWC was restored to levels similar to those of control plants.

### OJIP Curves

**FIGURE 5:**
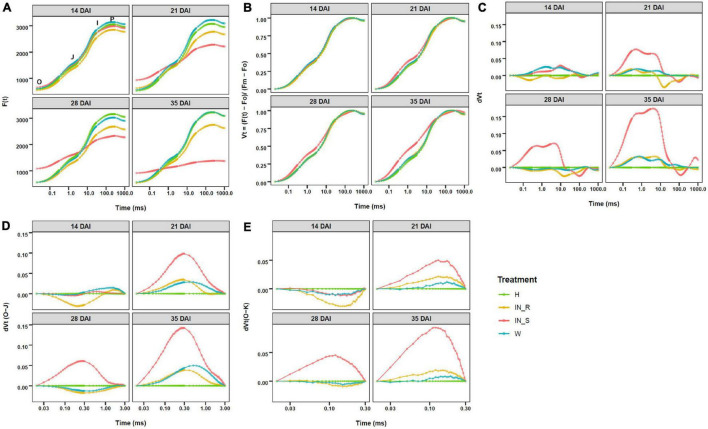
Fast chlorophyll a fluorescence (ChlF) OJIP curves (log time scale) recorded in dark-adapted needles of 22-month-old *Pinus pinaster* plants at 14, 21, 28, and 35 days after inoculation (DAI) with the pinewood nematode *Bursaphelenchus xylophilus*. Fluorescence values are expressed as: **(A)** raw ChlF transient curves; **(B)** relative variable fluorescence double normalized between F_0_ and maximum fluorescence (F_m_); **(C)** changes in O-P phase relative variable fluorescence intensity; **(D)** detail on changes in O-K phase relative variable fluorescence intensity; **(E)** detail on changes in O-K phase relative variable fluorescence intensity. Treatments were grouped in non-inoculated (healthy, H) mock-inoculated (wounded, W), susceptible PWN-inoculated plants (IN_S) and resistant PWN-inoculated plants (IN_R).

The OJIP curves described the ChlF rise from O (F_0_) to P (F_m_), with two intermediate steps; namely, J (F_J_ at ca. 3 ms) and I (F_I_ at ca. 30 ms). In general, the OJIP curves of non-inoculated and mock-inoculated control plants presented the typical three phase kinetics (O-J, J-I, and I-P) (Figures 5A,B). In comparison, OJIP curves of PWN inoculated plants showed a significant increase in F_0_ in susceptible plants and a significant decrease in F_m_ in susceptible and resistant plants, with exception of susceptible plants at 14 DAI and resistant plants at 28 DAI (Figure 5A and Supplementary Tables 3–5). The severe decrease in ChlF in susceptible plants strongly affected the shape of the OJIP curves (Figure 5A). In addition, double normalized OJIP curves showed that the largest deviations in relative fluorescence occurred in susceptible plants at the J step (V_J_), with a progressive increase starting at 21 DAI (Figure 5B). Further analysis of the ΔV_t_ curves revealed positive bands for susceptible plants at 21, 28 and 35 DAI, from O to the I-P phase, indicating differences in electron transport rates when compared to resistant and control plants (Figure 5C). A closer analysis of the differential curves for the O-J phase confirmed the existence of an intermediate K-band between F_0_ and F_J_ (ca. 0.3 ms) and a L-band between F_0_ and F_K_ (ca. 0.15 ms) in susceptible plants at 21, 28 and 35 DAI (Figures 5D,E).

### OJIP-Test Parameters

**FIGURE 6:**
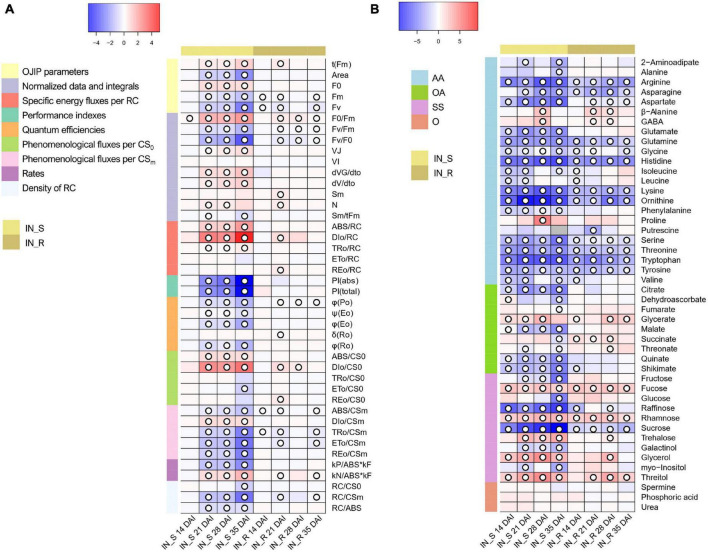
**(A)** Heatmap representing the changes in OJIP transients in dark-adapted needles of 22-month-old *Pinus pinaster* plants at 14, 21, 28, and 35 days after inoculation (DAI) with the pinewood nematode (PWN) *Bursaphelenchus xylophilus*. False color imaging was performed on log_10_-transformed data. Fold changes for PWN-inoculated plants (susceptible, IN_S, and resistant, IN_R) were calculated in relation to the mock-inoculated control for each time point (*n* = 15). Significant changes using Kruskal-Wallis test are indicated as 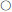 *p* < 0.05, with a false discovery rate (FDR) correction. **(B)** Heatmap representing the changes in relative levels of primary metabolites in stem tissues of 22-month-old *Pinus pinaster* plants 14, 21, 28, and 35 days after inoculation (DAI) with the pinewood nematode *Bursaphelenchus xylophilus*. Relative values (as means of 15 independent measurements) were normalized to the internal standard and fresh weight of the samples; false color imaging was performed on log_10_-transformed data. Fold changes for PWN-inoculated plants (susceptible, IN_S, and resistant, IN_R) were calculated in relation to the mock-inoculated control. Significant changes using one-way ANOVA are indicated as 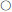 *p* < 0.05, with a false discovery rate (FDR) correction. Gray-color squares represent not detected (n.d.) values. Metabolites grouped in amino acids & derivatives (AA), organic acids (OA), sugars & sugar alcohols (SS), and others (O).

The effect of PWN inoculation on the photosynthetic apparatus of *P. pinaster* plants caused significant variations in several OJIP-test parameters (Figure 6A and Supplementary Tables 3, 4). No significant differences were found when comparing mock-inoculated and non-inoculated controls (Supplementary Table 5). Most significant changes were observed in susceptible plants at 21, 28 and 35 DAI. In these plants, and according to the OJIP curves, the complementary area between F_0_ and F_m_ (Area) which represents the electron transport from PSII RC to the quinone pool, significantly decreased. The time needed to reach Fm (t_Fm_) significantly increased and the time needed to obtain total RC closure (S_m_/t_Fm_) significantly decreased. F_v_/F_0_, considered as an indicator of the number and size of active photosynthetic RCs, significantly decreased from 14 DAI. The total number of electrons transferred to the electron transport chain (N) significantly increased at 21 and 28 DAI. A significant reduction in the number of active RCs (RC/ABS and RC/CS_m_) was supported by a significant increase in the rate of RCs closure (dV/dt_0_), absorbed energy per active RC (ABS/RC) and trapping per active RC (TR_0_/RC). Electron transport per CS_0_ (ET_0_/CS_0_) significantly decreased at 35 DAI. A strong significant increase in heat dissipation DI_0_/RC, DI_0_/CS_0_ and DI_0_/CS_m_ (up to 18-fold) and in maximum quantum yield of non-photochemical de-excitation (F_0_/F_m_) were observed at 35 DAI. Accordingly, the photochemical de-excitation rate constant (kP/ABS.kF) significantly decreased, whereas the non-photochemical de-excitation rate (kN/ABS.kF) significantly increased. The quantum efficiencies of electron transfer process (φ_Eo_, ψ_Eo_, and φ_Ro_) significantly decreased as well as the energy fluxes per CS_m_ (ABS/CS_m_, TR_0_/CS_m_, ET_0_/CS_m_, and RE0/CS_m_). The maximum photochemical efficiency (F_v_/F_m_ or φ_Po_) significantly decreased but the strongest significant reduction was observed in PI_TOTAL_ and PI_ABS_ at 35 DAI.

In resistant plants, F_m_ significantly decreased, except at 28 DAI. The normalized area above the OJIP curve which indicates the energy needed to close all RCs (S_m_) and the electron flux leading to the reduction of PSI (RE_0_/RC, RE_0_/CS_0_ and δ_Ro_) significantly increased at 21 DAI. As observed for susceptible plants, t_Fm_ and N also significantly increased at 21 DAI. A significant reduction in RC/CS_m_ was observed in resistant plants at 21 and 35 DAI. Heat dissipation (DI_0_/RC and DI_0_/CS_0_) significantly increased at 21 and 21-28 DAI, and F_0_/F_m_ significantly increased at 21, 28 and 35 DAI. Accordingly, the non-photochemical de-excitation rate significantly increased. Energy fluxes per CS_m_ significantly decreased (ABS/CS_m_ and TR_0_/CS_m_, at 14, 21 and 35 DAI, and ET_0_/CS_m_ and RE_0_/CS_m_ at 21 and 35 DAI). The F_v_/F_m_ and F_v_/F_0_ significantly decreased at 21, 28 and 35 DAI.

### Gas Chromatography Coupled to Time-of-Flight Mass Spectrometry Primary Metabolite Profiling

Gas chromatography coupled to time-of-flight mass spectrometry (GC-TOF-MS) analysis allowed the identification of 46 primary metabolites in stem tissues of *P. pinaster*; namely, 22 amino acids, 11 sugars and sugar alcohols, nine organic acids, and four other primary metabolites (Figure 6B and Supplementary Tables 6, 7).

When compared to non-inoculated controls, no significant changes were observed in the levels of primary metabolites of plants subjected to a mock inoculation (Supplementary Table 7). This indicates that the wound caused by the inoculation process had no significant impact in the primary metabolism of *P. pinaster* plants.

Overall, PWN-inoculated susceptible plants showed a significant decrease in the levels of most amino acids, including the branched-chained amino acids (BCAA) leucine, isoleucine and valine, and a significant increase in proline (up to 21-fold at 28 DAI), γ-aminobutyric acid (GABA) and β-alanine (up to 4-fold at 28 DAI) (Figure 6B and Supplementary Table 6). A general significant increase was observed for most sugars; namely, glycerol (up to 15-fold at 28 DAI), fucose (up to 4-fold 28 DAI), rhamnose (up to 6-fold 28 DAI), trehalose (up to 9-fold at 35 DAI), and threitol (up to 13-fold at 28 DAI). However, a significant decrease was observed in the levels of raffinose and sucrose after PWN inoculation. Organic acids also showed an overall significant decrease; namely, dehydroascorbate, quinate, shikimate and the tricarboxylic acid (TCA)-cycle intermediates citrate, malate and succinate.

The primary metabolite profile of resistant plants differed from that of susceptible plants, with less significant changes observed in the levels of several primary metabolites; namely, alanine, citrate, fructose, galactinol, glucose, glutamate, malate, *myo*-inositol, phenylalanine, and quinate (Figure 6B and Supplementary Table 6). However, similarly to susceptible plants, arginine, asparagine, aspartate, glutamine, glycine, histidine, lysine, ornithine, serine, threonine, tryptophan and tyrosine showed a significant decrease, whereas fucose, rhamnose, and threitol showed a significant increase (up to 8-fold at 28 DAI). A significant increase in the TCA-cycle intermediate succinate (up to 3-fold) was also observed at 14, 21 and 28 DAI. Other commonly known osmolytes also significantly increased in resistance plants, such as GABA (up to 2-fold at 21 and 28 DAI), glycerol and trehalose (up to 6-fold at 28 DAI).

**FIGURE 7:**
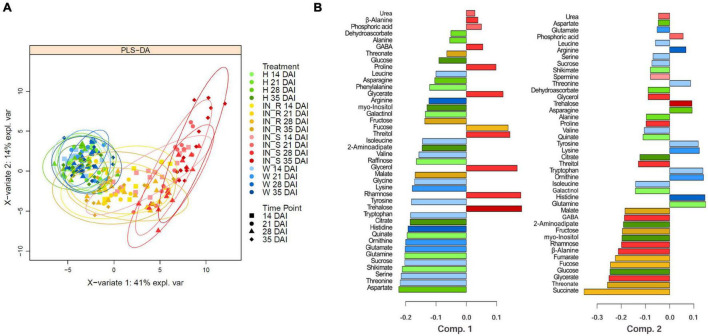
Partial least square discriminant analysis (PLS-DA) score **(A)**, and contribution plots **(B)** of the primary metabolite profile of stems of 22-month-old *Pinus pinaster* plants after inoculation with the pinewood nematode *Bursaphelenchus xylophilus*. Treatments were grouped in non-inoculated (healthy, H) mock-inoculated (wounded, W), susceptible PWN-inoculated plants (IN_S) and resistant PWN-inoculated plants (IN_R). DAI indicate days after inoculation. The bar length in the contribution plots represents the loading weights of each metabolite in component 1 and 2, respectively. The color indicates the sample group in which the metabolite has a maximal importance (based on the mean).

Supervised PLS-DA was applied to GC-TOF-MS primary metabolite data to identify major sources of variation in the data. The PLS-DA score plot of the first two components (PC1 and PC2) showed control groups (non-inoculated and mock-inoculated) clustered together and clearly separated from susceptible samples, particularly by the PC1 which explained 41 % of the total variance (Figure 7A). Resistant samples are scattered between the cluster formed by the control groups and the susceptible samples, overlapping mostly with susceptible samples collected 14 DAI and control samples. For all treatments, no separation between time points could be observed. The contribution plots (Figure 7B) further highlighted the importance of every metabolite for each component, based on the maximum mean relative abundance and respective group category. In general, the metabolites that showed a significant variation (*p* < 0.05) were those that mostly contributed to cluster separation. Control groups (non-inoculated and mock-inoculated plants) presented higher levels of most amino acids that significantly decreased in susceptible samples. In addition, β-alanine, GABA, glycerate, glycerol, proline, rhamnose, and threitol contributed the most for the clustering and separation of susceptible samples 28 DAI from the remaining treatments, while trehalose contributed the most for the separation of susceptible plants 35 DAI. On the other hand, fumarate, malate, succinate, threonate, fructose and fucose were the metabolites that most promoted the clustering of resistant samples (particularly succinate). The remaining metabolites (mostly amino acids) contributed for the clustering of control groups.

### Integration of Metabolomics and Physiological Data

**FIGURE 8:**
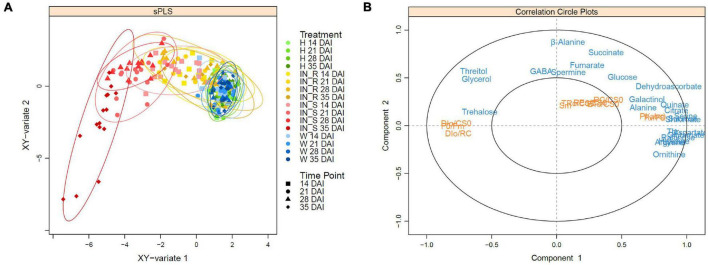
Sparse partial least squares (sPLS) regression analysis score plot **(A)** and correlation circle plot **(B)** of the datasets of OJIP transients and primary metabolite profiling in stem tissues of 22-month-old *Pinus pinaster* plants, 14, 21, 28, and 35 days after inoculation (DAI) with the pinewood nematode *Bursaphelenchus xylophilus*. Primary metabolite data (blue) were used as predictor for physiological responses (orange). Treatments were grouped in non-inoculated (healthy, H) mock-inoculated (wounded, W), susceptible PWN-inoculated plants (IN_S) and resistant PWN-inoculated plants (IN_R).

Sparse PLS (sPLS) analysis highlighted the differences between the *P. pinaster* response profiles after PWN inoculation (Figure 8A). A clear separation between susceptible and control plants (i.e., non-inoculated and mock inoculated) was observed on the horizontal axis (x-axis). Sparse PLS analysis further highlighted the separation of the susceptible plants at 35 DAI from all the other clusters. Resistant plants were clustered between susceptible and control plants. The cluster of resistant plants at 35 DAI overlapped with the control plants, revealing a high correlation between these sample groups.

The sPLS correlation circle plot revealed a high number of highly correlated metabolites on the positive side of the x-axis, and together with the physiological parameters PI_ABS_ and F_v_/F_0_, were responsible for the cluster of control and resistant samples at 35 DAI groups (Figure 8B). On the negative part of the x-axis, sugars glycerol, threitol, and trehalose, and the physiological parameters DI_0_/CS_0_, DI_0_/RC and F_0_/F_m_, whose levels significantly increased with PWN infection, were responsible for the separation of the susceptible plants at 35 DAI. On the positive side of the y-axis, β-alanine, GABA, and spermine were responsible for the cluster of the remaining susceptible plants (up to 28 DAI) whereas TCA-intermediates fumarate and succinate were the metabolites most responsible for the cluster of resistant plants.

## Discussion

### Intraspecific Variation of Anatomical Defenses in *Pinus pinaster* Affects Susceptibility to Pine Wilt Disease

The anatomical observations of PWN-inoculated susceptible *P. pinaster* plants are in agreement with previous reports for other susceptible species of the Pinaceae family (Mamiya and Shoji, 2009; Nunes da Silva et al., 2013, 2015). Once inside the plant, the nematodes disperse to the cortex, cortical resin ducts, cambium, xylem axial and radial resin ducts, finally reaching the pith at an advanced PWD stage. The nematodes use the resin ducts to migrate vertically, reproduce, and feed on the epithelial cells lining the ducts (Son et al., 2010). These anatomical modifications lead to PWD symptoms, mainly due to disruption of water transport (Fukuda, 1997). However, in resistant *P. pinaster* plants tissue damage was restricted, presumably to the inoculation site. The observed PWN distribution in compartmentalized areas in resistant plants is likely related with highly lignified cell walls surrounding cambial cavities and resin ducts (Kusumoto et al., 2014). In agreement, gene expression analysis, using the same *P. pinaster* half-sib family, revealed an over-expression of genes involved in lignin synthesis in resistant plants 72 h after infection, which translated to an increase in lignin content and cell wall lignification in stem tissues around the inoculation zone in these plants (Modesto et al., 2021). This modification hinders the PWN horizontal migration and delays damage expansion in the cortex and xylem.

Despite the increase in cell wall lignification in resistant *P. pinaster* plants, primary metabolite profiling did not show an accumulation of phenylalanine, up to 35 DAI. Phenylalanine is the first precursor of the phenylpropanoid pathway which is involved in the production of lignin, among other important secondary metabolites involved in plant defense (e.g., flavonoids, lignans) (Dixon et al., 2002; Bhuiyan et al., 2009; Tohge et al., 2013). No significant increase in phenylalanine levels suggests that the increase in lignin biosynthesis and cell wall lignification in resistant plants occurred in the early hours/days after PWN infection. These results are in agreement with the previous reports of lignin accumulation occurring mainly in the first days after PWN infection in resistant Pinaceae species such as *Picea abies* (Nunes da Silva et al., 2013), and resistant *P. pinaster* plants (Modesto et al., 2021). In *P. abies* by 14 DAI lignin content had already significantly decreased and for resistant *P. pinaster* plants no data was collected at later days after infection.

The observed occlusion of resin ducts with a gel-like resin in resistant plants has also been reported in *P. thunbergii* as an additional resistance mechanism to prevent the vertical migration of the nematodes (Kusumoto et al., 2014). The reduction in PWN migration and multiplication ultimately allows host plants to survive PWN infection (Fukuda, 1997; Nunes da Silva et al., 2013).

In addition to the intrinsic differences between the PWN-inoculated plants, it should be highlighted that the differential susceptibility to PWN observed in this study could also be due to failures of the inoculation process. Despite the large number of PWN often used in inoculation experiments (up to 10000), only ca. 10 % of the inoculum is estimated to successfully invade the plant tissue (Kuroda, 2008). In susceptible species, the number of nematodes inside the plant rapidly increase resulting in larger numbers than those initially inoculated (Nunes da Silva et al., 2014, 2015; Menéndez-Gutiérrez et al., 2018); for initial concentrations of at least 50 PWN/plant, the success of the inoculation procedure and plant mortality has been reported to be over 92 % (Abelleira et al., 2013).

In this study, because the classification of plant resistance was based solely on external symptoms after PWN-inoculation, microscopy analysis of randomly selected susceptible and resistant plants allowed to confirm the presence of the PWN and/or cellular damage caused by the nematode migration. These observations showed that the nematodes successfully entered the plants through the wounds, thus validating the success of the inoculation process.

### Pinewood Nematode Infection Impairs Photosynthesis in Susceptible *Pinus pinaster*

The OJIP curve represent the efficiency of electron transfer in the intersystem chain, from the reduction of the primary acceptor of PSII to the end electron acceptors at the PSI acceptor side. Any deviation from its typical shape is an indication of photosystem malfunction (Strasser et al., 2004; Goltsev et al., 2016; Kalaji et al., 2016; Baghbani et al., 2019). In this study, the evaluation of the OJIP curves observed in susceptible plants suggested that susceptible plants are more prone to damage to the photosynthetic apparatus upon PWN infection, in comparison with resistant plants. These changes include the appearance of L- and K- bands, well-known indicators of environmental stress (Kalaji et al., 2016). The K-band is related to damage in the oxygen evolving complex (OEC) due to an imbalance between the electron flow from the OEC to the RC at acceptor site of PSII, leading to a progressive decrease in the rates of photochemical processes (Strasser et al., 2004; Kalaji et al., 2016; Baghbani et al., 2019). The enhanced L-band is related with a decrease in the excitation energy transfer between PSII units (i.e., PSII connectivity) (Yusuf et al., 2010). The OJIP transients were used to evaluate the electron transport flux from PSII RCs to PSI. Changes in OJIP transients (e.g., Area, F_0_, V_J_) in susceptible plants, indicated a blockage in electron transfer from the RC to the quinone pool. When primary quinone Q_A_ is unable to accept electrons from PSII, most likely due to inactivation of OEC, the RCs are converted to inactive. In addition, the observed increase in ABS/RC, and TR_0_/RC (i.e., enhanced efficiency per RC), did not result in the increase of electron transport energy ET_0_/RC, but in a sharp increase in DI_0_/RC, implying that most energy was dissipated as heat (Maxwell and Johnson, 2000). The inactivation of RCs is considered a down-regulation mechanism to dissipate the excess of absorbed light and confer protection against photo-oxidative stress (Stirbet and Govindjee, 2011; Monneveux et al., 2013; Kalaji et al., 2014; Meng et al., 2016). In susceptible plants, the reduction in the flux ratio of PSI and PSII was increasingly severe in response to disease progression, whereas the enhanced energy flux of PSI (δ_Ro_) in resistant plants suggested an increased tolerance of PSI to stress (Gupta, 2019).

The PSII functional status is commonly evaluated by the F_v_/F_m_ ratio. However, because F_v_/F_m_ is considered mostly unresponsive to drought stress, F_v_/F_0_ has been suggested to be a preferable alternative to assess photosynthetic capacity (Maxwell and Johnson, 2000; Mohammed et al., 2003; Oukarroum et al., 2007). In PWN-inoculated plants, both parameters, particularly F_v_/F_0_, confirmed the down-regulation of PSII and impairment in the water-splitting efficiency in PWN-inoculated plants, as previously reported for other environmental stress responses (Goltsev et al., 2016; Kumar et al., 2020).

The PI is a widely used parameter to quantitatively assess plant vitality. The three PI_ABS_-dependent parameters were drastically decreased in susceptible plants; namely, (i) RC density (RC/ABS), (ii) quantum yield of primary photochemistry (φ_P0_), and (iii) electron transport beyond Q_A_ in PSII (ψE0) (Strasser et al., 2004, 2010; Stirbet and Govindjee, 2011; Banks, 2017). PI_TOTAL_ further includes information of the reduction of the end electron acceptors at the PSI acceptor side (δ_Ro_), and therefore, allows to quantitatively assess the integral functional activity of PSII, PSI, and intersystem electron transport chain. Because δ_Ro_ remained mostly unchanged in susceptible plants, PI_TOTAL_ was influenced by PI_ABS_ parameters, indicating a general down-regulation of PSII function (Silvestre et al., 2014; Ceusters et al., 2019).

The decrease in photosynthetic activity was most likely induced by a water deficiency in the leaves, as confirmed by the decrease in the RWC in susceptible plants. Previous physiological studies in PWN-inoculated *Pinus* spp. reported that the disruption of the water conductance, caused by xylem embolism, led to a decrease in leaf water potential, inactivation of the water-splitting complex of PSII, and ultimately, to the cessation of photosynthetic activity (Melakeberhan et al., 1991; Fukuda, 1997; Gao et al., 2017; Menéndez-Gutiérrez et al., 2017). The decrease in energy production, crucial to drive carbon fixation, negatively affects energy demanding processes related to growth and plant defense, thus contributing to plant susceptibility to PWN (Melakeberhan and Webster, 1990). On the other hand, this study showed that, by restricting PWN infection, resistant plants were able to maintain photosynthetic function without major significant changes in OJIP transients, and recover from a decrease in RWC to levels close to control plants at 35 DAI.

### Primary Metabolite Adjustments Influence *Pinus pinaster* Resistance to Pinewood Nematode

Pinewood nematode (PWN) infection caused local changes in the primary metabolite levels of *P. pinaster*. The significant decrease in sucrose in PWN-inoculated plants is consistent with reports of enhanced activity of the cell wall invertase that cleaves sucrose into glucose and fructose at the infection site. The accumulation of these metabolites is further associated with induced expression of pathogenesis-related genes (Proels and Hückelhoven, 2014; Rojas et al., 2014). However, as PWD progressed, the levels of glucose and fructose decreased in susceptible plants. Sugar accumulation is highly affected by changes in photosynthetic activity and metabolite uptake (e.g., hexoses) by the pathogen, e.g., during the phytophagous stage of the PWN (Berger et al., 2007; Kanwar and Jha, 2019).

The levels of most amino acids were also significantly reduced, including BCAAs which are usually accumulated in response to osmotic stress (Persson et al., 2016). In the absence of photosynthesis, energy demand under stress conditions can promote the degradation of BCAAs to generate additional energy from protein degradation (Caldana et al., 2011; Less et al., 2011). Photosynthetic impairments could also explain the decrease in phenylalanine in susceptible plants up to 35 DAI. In trees, a large amount of the fixed carbon is channeled through phenylalanine for the activation of the shikimate pathway and the biosynthesis of phenylpropanoids, including flavonoids, stilbenes, coumarins, SA, tannins, and lignin (Pascual et al., 2016; Abreu et al., 2020). In contrast, studies on *P. pinaster* responses in the first hours after PWN infection showed an over-expression of genes involved in phenylpropanoid biosynthesis, including defense-related flavonoid and lignin biosynthesis (Modesto et al., 2021). The observed decrease in phenylalanine at an advanced stage of PWN infection suggests a decrease in the production of these defense-related secondary metabolites in susceptible plants, and a consequent lack of enhanced cell wall lignification as an inducible physical defense strategy.

The observed accumulation of osmolytes (e.g., fucose, GABA, proline, rhamnose, threitol, and trehalose) contributes to the maintenance of cell turgor through a decrease in the osmotic potential and protection against oxidative damage by restoring cellular redox balance (Krasensky and Jonak, 2012; Arbona et al., 2013; Jorge et al., 2016). In addition to its role as osmolyte, the accumulation of GABA has been suggested to play a role in signaling and regulatory mechanisms of stress responses (Michaeli and Fromm, 2015; Carillo, 2018; Ramos-Ruiz et al., 2019). The observed accumulation of alanine, GABA and succinate, particularly in resistant plants at 21 and 28 DAI, suggests the activation of alanine metabolism and the GABA shunt (i.e., a bypass pathway of the TCA cycle from 2-oxoglutarate *via* succinyl-CoA to succinate) (Fait et al., 2008). sPLS and PLS-DA analysis further confirmed the strong contribution of succinate to the clustering and separation of resistant plants from the remaining treatments. Alanine metabolism and the GABA shunt are activated at the expense of the production of other amino acids (e.g., asparagine, aspartate, glutamate, glutamine, glycine, serine), thus presumably explaining the observed general decrease in amino acids. Increased GABA-shunt activity has been associated with plant resistance due to the regulation of C:N metabolism and hypersensitive responses (HR), to ultimately delay infection-induced senescence (Seifi et al., 2013; Michaeli and Fromm, 2015; Ramos-Ruiz et al., 2019). A comparison of transcriptional changes associated with PWN infection in *P. thunbergii* revealed that tree resistance likely relies on a moderated and regulated HR and cell wall fortification to restrict PWN migration. On the contrary, fast-induced transcripts in susceptible plants led to a HR response that ultimately caused tree death (Hirao et al., 2012). A similar observation has been reported for defense-related phytohormones showing a significant increase only in susceptible *P. pinaster* plants as part of an ineffective trigger of the HR (Rodrigues et al., 2021). Immuno-localization of GABA in vascular tissues of pine seedlings (e.g., differentiating xylem, ray parenchyma, and epithelial resin duct cells) further highlights the role of this metabolite not only in vascular processes but also in inducible defense mechanisms, including cell wall lignification (Molina-Rueda et al., 2015).

Defense responses in plants require a large supply of energy, mainly from primary metabolism (e.g., TCA-cycle intermediates) (Berger et al., 2007; Bolton, 2009). In parallel with the downregulation of processes associated with plant growth (e.g., photosynthesis), this energy can be diverted and used for defense responses or for further energy production (Rojas et al., 2014; Kanwar and Jha, 2019). During the host-pathogen interaction, the GABA-shunt activation might help to support the costly defense-related metabolic pathways through the up-regulation of the TCA cycle for energy production, feeding succinate and NADH to maintain respiration (Bolton, 2009; Michaeli and Fromm, 2015). Gao et al. (2017) has reported an increase in the respiration rate in *P. massoniana* upon PWN infection, after the first external symptoms, probably to meet the higher energy demand to support defense responses. In this study, the observed accumulation of succinate in resistant plants might also be explained by its use as electron donor to the respiratory system.

One early symptom of PWD is the cavitation and embolism of the xylem, ultimately leading to a blockage of the water-conducting system (Futai, 2013). Thus, it is possible that oxygen transport *via* the xylem sap is also hindered, leading to a hypoxic environment in stem tissues (Gansert, 2003; Wittmann and Pfanz, 2014). This would also explain the observed accumulation of alanine, GABA and succinate, which are well-known products of hypoxic metabolism in oxygen-deprived plant tissues (van Dongen et al., 2009; Rocha et al., 2010; Zabalza et al., 2011; António et al., 2016). The extent of oxygen deprivation in stem tissues of *Pinus* spp. after PWN infection is still unknown and would be relevant to investigate. The use of stable isotope labeling experiments (^13^C and ^15^N) would provide further evidence for the possible activation of hypoxia-related metabolic pathways (António et al., 2016). Hypoxia mechanisms in plants have been comprehensively described in response to waterlogging, but during the last decade, these studies have been extended to the involvement of hypoxia in plant development (Weits et al., 2020) and plant–pathogen interactions (Loreti and Perata, 2020; Valeri et al., 2020). In response to pathogen infection, local hypoxia might be required to activate specific oxygen-sensitive resistance pathways, or it might affect the production of elicitor molecules (Loreti and Perata, 2020).

## Concluding Remarks

Overall, this work provides new insights into the physiological changes associated with *P. pinaster* differential responses to PWN infection. The integration of photosynthetic and metabolic data allowed to identify specific traits possibly associated with *P. pinaster* resistance to PWN, as a valuable resource for future studies. In general, susceptible plants were characterized by an increase in osmolytes (e.g., trehalose and threitol) and in heat dissipation as a protection mechanism against photo-oxidative stress. On the other hand, resistant plants did not show major significant changes in ChlF parameters revealing no photosynthetic impairments after PWN infection. The significant increase in GABA shunt-related metabolites suggests a possible role of these metabolites in *P. pinaster* resistance to PWN and should be further explored. Additional stable isotope labeling experiments coupled to GC-TOF-MS analysis would allow to derive a comprehensive and quantitative overview of the central carbon and nitrogen metabolic network during the time course of PWN infection. Altogether, results from this study provide interesting opportunities for future research on PWD and resistance traits in *Pinus* spp. with particular relevance in modern plant breeding programs and PWD management strategies. Indeed, metabolomics provides a key link between the genotype and phenotype, and is particularly useful for the prediction of complex traits (Fernie and Schauer, 2009; Alseekh and Fernie, 2021; Fernandez et al., 2021). Furthermore, the integration of metabolomics with other omics data can greatly accelerate candidate gene identification and comprehensive metabolic pathway elucidation, ultimately leading to biomarker discovery and plant improvement.

## Data Availability Statement

The raw data supporting the conclusions of this article will be made available by the authors, without undue reservation.

## Author Contributions

AR, CA, and IC conceptualized and designed the study. AR performed all experiments, statistical analysis, and wrote the first draft of the manuscript. IC defined the experimental design, provided greenhouse resources, and assisted in PWN inoculation assay and statistical analysis. CA secured funding and supervised the research. All authors reviewed and approved the submitted version of the manuscript.

## Conflict of Interest

The authors declare that the research was conducted in the absence of any commercial or financial relationships that could be construed as a potential conflict of interest.

## Publisher’s Note

All claims expressed in this article are solely those of the authors and do not necessarily represent those of their affiliated organizations, or those of the publisher, the editors and the reviewers. Any product that may be evaluated in this article, or claim that may be made by its manufacturer, is not guaranteed or endorsed by the publisher.

## Footnotes

1
http://gmd.mpimp-golm.mpg.de/
