# Supplementary material for: Primary Metabolite Adjustments Associated With Pinewood Nematode Resistance in Pinus pinaster
Source: Front Plant Sci. 2021 Nov 24;12:777681. doi: 10.3389/fpls.2021.777681 (PMC8691400; doi:10.3389/fpls.2021.777681)
Supplement: Supplementary file 1 [file Data_Sheet_1.PDF]

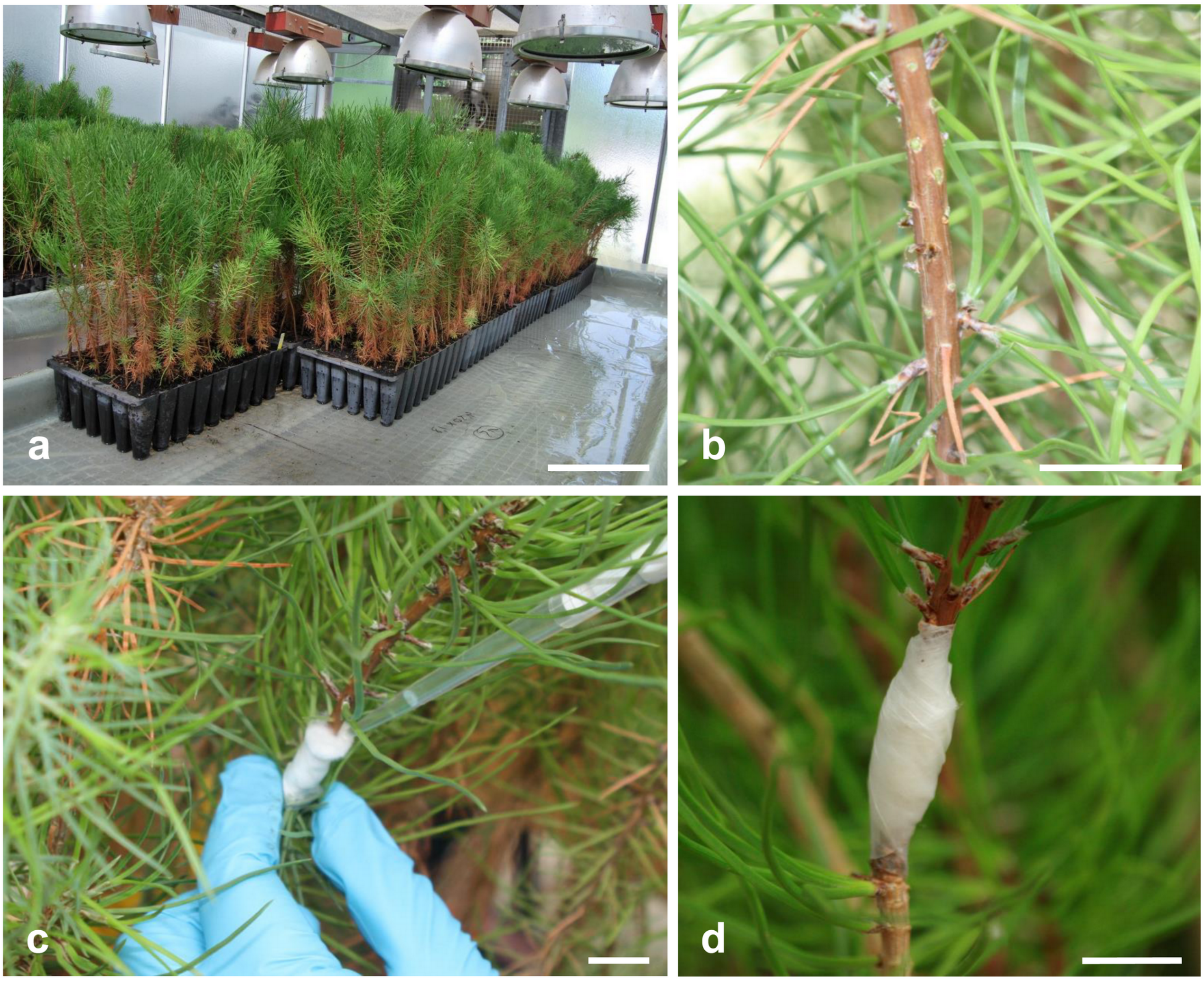

Supplementary Figure 1. Pinewood nematode *Bursaphelenchus xylophilus* inoculation procedure in 22-month-old *Pinus pinaster* half-sib family. (**a**) general aspect of *P. pinaster* plants before inoculation; (**b**) needles were removed from an area of ca. 5 cm in the upper part of the stem of each plant, and longitudinal cuts were performed with a razor blade; (**c**) a sterilized cotton was fixed to the inoculation area with Parafilm® and soaked with 500 µL of a suspension of ca. 1000 PWN mL^{-1} in distilled water; (**d**) The cotton was sealed with Parafilm® to prevent inoculum from drying. Scale bars = 30 cm (a), 2 cm (b-d).
